# Research on Lane Changing Game and Behavioral Decision Making Based on Driving Styles and Micro-Interaction Behaviors

**DOI:** 10.3390/s22186729

**Published:** 2022-09-06

**Authors:** Ming Ye, Pan Li, Zhou Yang, Yonggang Liu

**Affiliations:** 1 Key Laboratory of Advanced Manufacturing Technology for Automobile Parts, Ministry of Education, Chongqing University of Technology, Chongqing 400054, China; 2 State Key Laboratory of Mechanical Transmission, College of Mechanical and Vehicle Engineering, Chongqing University, Chongqing 400044, China

**Keywords:** autonomous vehicle, game theory, path planning and tracking, lane change, driving risk field

## Abstract

Autonomous driving technology plays an essential role in reducing road traffic accidents and ensuring more convenience while driving, so it has been widely studied in industrial and academic communities. The lane-changing decision-making process is challenging but critical for ensuring autonomous vehicles’ (AVs) safe and smooth maneuvering. This paper presents a closed-loop lane-changing behavioral decision-making framework suitable for AVs in fully autonomous driving environments to achieve both safety and high efficiency. The framework is based on a complete information non-cooperative game theory. Moreover, we attempt to introduce human driver-specific driving styles (reflected by aggressiveness types) and micro-interaction behaviors for both sides of the game in this model, enabling users to understand, adapt, and utilize intelligent lane-changing techniques. Additionally, a model predictive control controller based on the host-vehicle (HV) driving risk field (DRF) is proposed. The controller’s optimizer is used to find the optimal path with the lowest driving risk by using its optimizer and simultaneously adjusting its control variables to track the path. The method can synchronize path planning and motion control and provide real-time vehicle state feedback to the decision-making module. Simulations in several typical traffic scenarios demonstrate the effectiveness of the proposed method.

## 1. Introduction

### 1.1. Introduction and Related Work

As an essential component of autonomous driving technology, the automatic lane-changing system (ALCS) has attracted the interest of numerous scholars worldwide [1]. In ALCS, the decision-making module is responsible for generating the driving behaviors of the vehicle, which connects environmental perception, trajectory planning, and motion control; it is the most fundamental and critical component of AVs intelligence [2].

Many scholars around the world have conducted extensive and in-depth research on the decision-making model of autonomous driving in recent years. One of the primary bottlenecks of autonomous driving decision-making algorithms has always been how to make vehicles make reasonable and correct decisions in a complex environment full of uncertainties. Furda and Vlacic use a multi-criteria decision-making algorithm to select the optimal driving strategy based on the candidate action set [3]. The Markov decision process (MDP) is a common model that studies the probability of decision-making and can deal with decision-making uncertainty [4]. In addition, the technological innovation of machine learning algorithms has led to the rapid development of data-driven decision-making algorithms. Ulbrich established a vehicle lane-changing state assessment and vehicle decision-making judgment model based on the dynamic Bayesian network to achieve the optimal efficient lane-changing [5]. Li et al. [6] developed a data-driven deep neural network (DNN) driving algorithm to have an adaptive effect on road conditions in real-world traffic scenarios, but for DNN training and testing, a considerable number of high-quality data samples are required. Based on the reinforcement learning (RL) method, the authors integrate the reward mechanism of RL into the interaction of the stochastic MDP and the traffic environment, solve the sequential decision-making problem of the host vehicle, and effectively address the lack of training data in [7]. In addition to the learning-based lane-changing models previously mentioned, there are also expert system-based, fuzzy logic-based, and rule-based lane-changing decision-making methods [8,9,10]. Even though the above-mentioned traditional lane-changing models have been extensively studied and have yielded significant results, modeling and logical design challenges will arise due to interactions in complex traffic environments. Moreover, the model’s inflexibility and inability to adapt to different driving styles is a result of the static method of lane change decision-making.

Efforts have been made to study interaction-aware models in order to capture the interdependence between vehicles. Coupled HMM models (CHHMs) are used to model pairwise dependencies between multiple moving entities [11]. While this technique can tell when we change lanes, we did not go into detail about how. The study of decision-making problems involving interactions between vehicles can also be approached using game theory. In [12], a Stackelberg game-based lane-changing model is proposed by evaluating the aggressiveness of surrounding vehicles and incorporating it into the payoff function’s design. It can simulate the interaction behaviors between autonomous vehicles and human drivers with different aggressiveness types in mixed-traffic scenarios. Wang et al. [13] proposed a differential game-based vehicle tracking and lane change control model to prevent future situations that are detrimental to the revenue of the host vehicle. In [14], the authors used Stackelberg game theory to model the driver model of AVs and then used the model to simulate the driver’s reasoning process. Finally, the interaction behavior similar to the human driver is realized. Arbis et al. established a lane-changing model based on game theory, which represented the game process of lane-changing drivers and surrounding interacting vehicle drivers by using turn signals and continuous lateral displacement [15]. Aiming at the interaction of multiple vehicles in the lane-changing process, a lane-changing model including cooperative and game behaviors is established, which introduces and quantifies the interaction between multiple vehicles [16].

Although the above-mentioned decision-making research based on game theory can effectively deal with vehicle micro-interaction behaviors under complex traffic conditions, there is an important problem that has not been well considered. If the design of the decision-making algorithm lacks the real-time feedback of vehicle planning and motion control, it may lead to the lack of feasibility of the planned path. An unsuitable path or driving beyond the control constraints may result in frequent braking and sharp steering maneuvers on the planned path, deteriorating the driving safety and comfort dramatically.

Lower computational requirements allow for local path planning, maneuver planning, and motion control in dynamic environments. The driving risk field refers to the quantitative description of the driving risk faced by the vehicle using a mathematical model analogous to the “physical field” and based on dynamic traffic information obtained around the vehicle. This kind of research is developed from the trajectory planning method of mobile robots based on the concept of artificial potential field proposed by Khatib [17]. The method of using field theory to construct a driving risk field for driving safety assessment has also been extensively discussed with the advancement of the field of intelligent transportation. Wu Jian et al. [18] developed a unified model of the human-vehicle-road closed-loop system’s DRF based on different object attributes. Ji and Khajepour developed a three-dimensional virtual risk field as a trigonometric function about the road and an exponential function about the obstacles and gradient descent is used to generate the vehicle’s desired collision avoidance trajectory. However, the tracking control of trajectories and the problem of easily falling into local minima are not well considered in [19]. In [20], researchers built a risk field with a vehicle tire model that considered obstacles and road constraints.

These studies usually assume that surrounding vehicles have constant velocity or acceleration, and the impact of vehicle interaction on the real-time transition of the host vehicle’s state is not adequately considered. However, these assumptions are not always true in the real world, which can lead to uncomfortable and impractical lane changes. In terms of improving the maneuverability, safety, and driving stability of AVs, it is imperative to develop a path planning and control algorithm that can respond in real-time to vehicle decisions and account for interactions between vehicles.

Whether the host vehicle eventually decides to change lanes depends on the reactions of different types of drivers in the same situation, in addition to the fundamental parameters of the participating vehicles on the road (e.g., velocity, acceleration, position, etc.). In this paper, existing research is used to introduce the aggressiveness parameter (β), which effectively resolves this consideration. The aggressiveness type plays a crucial role in the decision-making process of participating vehicles in a dynamic game. A competing vehicle (CV) of high-aggressiveness (big β) prefers maintaining its velocity and positional advantage by not yielding to the host vehicle in a lane-changing scenario [21] whereas less-aggressive CV (Small β) tends to yield to HV to ensure their own driving safety [22]. This article incorporates aggressiveness into the design of decision-making and path-planning algorithms. This consideration has the potential to promote the self-adaptation of AVs to different kinds of drivers and passengers, as well as the advancement of personalized driving technology, hence shifting the status quo from “people adapting to vehicles” to “vehicles adapting to people.”

### 1.2. Contribution

The main contributions of this paper are as follows. Firstly, based on the complete information non-cooperative game theory, a closed-loop lane-changing behavior decision model for AVs that takes into account the aggressiveness types and vehicle micro-interaction behaviors of both game participants is established. Then, beginning with the field theory, a unified risk description model for the driving environment is created, and the field strength distribution of the DRF is incorporated into the MPC controller cost function. On this basis, a novel coupled path planning and motion control DRF-based MPC controller is proposed. Finally, the feasibility and effectiveness of the algorithm are tested in several typical lane-changing scenarios.

### 1.3. Paper Organization

The remainder of this article is organized as follows. In Section 2, the system architecture of the closed-loop decision algorithm is outlined. In Section 3, a lane-changing decision model based on complete information non-cooperative game theory is established. In Section 4, an MPC controller incorporating the host-vehicle driving risk field is designed. In Section 5, we design two typical cases to verify the proposed algorithm and analyze and discuss the test results. Finally, Section 6 concludes the paper.

## 2. Problem Formulation and System Construction

**Figure 1:**
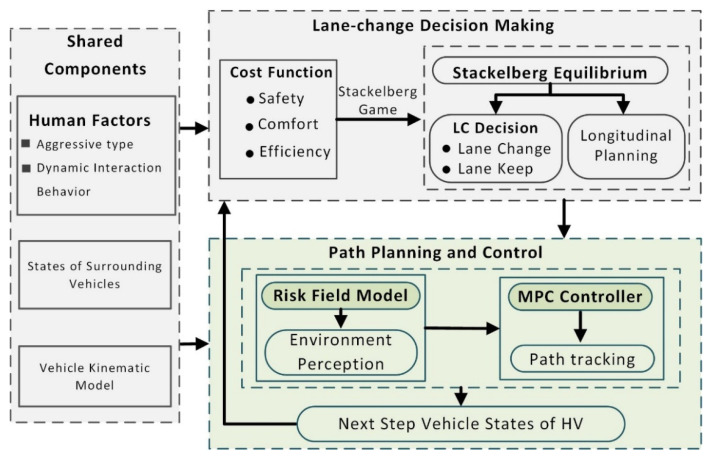
The lane-changing behavior decision-making framework for AVs.

In a typical freeway lane change scenario, the proposed lane-changing behavior decision framework is shown in Figure 1. The model is mainly comprised of three mutually coupled functional modules: decision-making, path planning, and motion control. In the decision-making module, cost function considering driving safety, travel efficiency, and driving comfort are established. Then, by solving the Stackelberg game, the optimal strategy, including the optimal lane-changing command and the optimal longitudinal acceleration, is obtained and then transmitted to the path planning and control module. Note that this paper combines the longitudinal planning of the HV with the lane-changing decision. Then, this paper completes the unified risk perception of the road environment based on the established DRF model. The road environment is composed of traffic entities, including surrounding obstacle vehicles (OVs), road boundaries, and lane lines. The future state of the HV can be predicted using MPC. To take the risk field strength of the predicted state as a key component of its cost function, MPC used its optimizer to help find the optimal driving path with the lowest risk while simultaneously tracking the path by adjusting control output. Note that this controller can not only solve the problem that the conventional artificial potential field method is prone to local minima but also can tackle the control problem of multiple inputs and multiple outputs (MIMO). Finally, the module feeds back the planned path parameters and vehicle states to the decision-making module to address the decision-making at the next time step.

This paper defines the aggressiveness parameter as the degree of preference of different driving groups for travel efficiency in an effort to allow the decision-making of autonomous vehicles to be smarter and humanized. That is, vehicles with higher aggressiveness values have higher velocity expectations during lane changes and strive for larger driving spaces while being capable of withstanding higher levels of road risk. The aggressiveness types of drivers are generally divided into three types (conservative, moderate, and aggressive). Each type of driving style differs considerably in aggressiveness value and travel efficiency preferences. When the aggressiveness value is high, e.g., AVs should behave like aggressive drivers, preferring higher expected velocity and acceleration, shorter following distances, and less concern for driving safety when performing lane changes. The assessment of risk in the driving environment has also become bolder. Aggressiveness is incorporated into the design of the decision cost function and DRF model in this paper.

In the method proposed in this paper, the following assumptions are made:

- It is assumed that the vehicles studied in this paper are all AVs and have been equipped with complete onboard sensors and wireless communication modules (i.e., V2V and V2I technologies) to obtain rich information about the surrounding vehicles motion status and road environment.
- Only the acceleration and deceleration behaviors of surrounding vehicles are considered, and their lane-changing behaviors are not considered.
- The vehicles studied are all cars, excluding other types such as trucks and motorcycles.

## 3. Mathematical Modeling of Lane-Changing Decision

Game theory is an effective approach to investigating the interactions of decision makers. A game is a mathematical object with well-defined elements, including players, strategy space, and payoffs [23]. This article examined the payoffs from the perspective of decision-making cost, and a complete information non-cooperative game theory approach is used to handle the lane-changing decision problem of AVs.

### 3.1. Game Formulation

**Figure 2:**
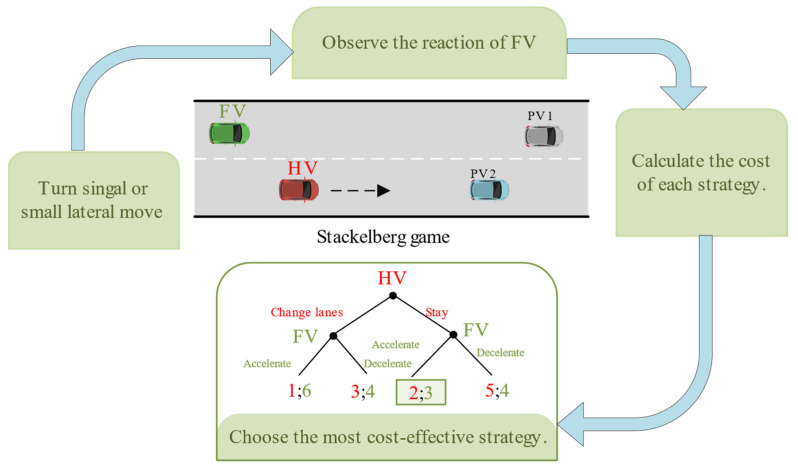
Stackelberg game theory.

As illustrated in Figure 2, the entire process mimics the conventional human driver’s lane-change decision and its corresponding longitudinal velocity adjustment strategy. When the preceding vehicles (PV) 2 is driving too slowly, the HV may consider changing lanes. Before doing that, the HV would evaluate the target lane’s traffic conditions using environmental perception information. If sufficient space is available, the HV will interact with the following vehicles (FV) by activating the turn signal or making a small lateral move to observe the FV’s reaction. It is presumed that in a few seconds, the FV reacts to the HV’s actions by the acceleration it might make. Note that if the aggressiveness types of the two sides of the game changed, the final strategies and decision-making costs of the players will also be different.

Figure 2 presents the decision costs for four interaction scenarios between HV and competing vehicle FV. With two players, HV is the leader who can choose either to change lanes or to keep lanes. FV is this follower; it has the option of yielding to HV based on the leader’s decision. Stackelberg game theory is used in this paper to simulate the multi-agent decision-making problem with sequential order. The Stackelberg game theory is used in this paper to mimic this multi-agent sequential decision-making problem. When HV chooses to change lanes, FV prefers to yield to HV, according to the illustration cost matrix in Figure 2. Likewise, HV selects lane keeping, and FV may accelerate appropriately based on the cost. In conclusion, both sides of the game evaluate and search for the decision cost corresponding to the strategy pairs to determine the optimal strategy pair (accelerate, stay). Therefore, we model the lane change decision-making as a two-player equilibrium game problem with different decision cost functions, where an equilibrium exists at all times.

The overall structure of the Stackelberg game is shown in Table 1, in which α denotes the player’s longitudinal acceleration and U represents the cost of the strategic combination. The HV not only decides whether to change lanes but also the optimal longitudinal acceleration required at the present time step. Since the acceleration is continuous, there are infinite combinations of strategies. Additionally, FV also has an infinite number of policy combinations; it can select any value within the constraint range, i.e., $\left(\alpha_{\min},\alpha_{\max}\right)$. Throughout the game, the players are trying to minimize their cost. Consequently, the decision cost function plays a crucial part in the generation of reasonable decisions.

### 3.2. Definition of Cost Function

The decision cost considers a combination of three factors in order to generate a reasonable decision logic for the host vehicle and the competing vehicle.

The first part $U_{ds}^{HV}$ quantifies the level of safety associated with vehicle lane-changing and lane-keeping. The second factor is $U_{te}^{HV}$, which evaluates the vehicle velocity and travel space benefits. The final component is $U_{dc}^{HV}$, which ensures the vehicle’s comfort during the game. The total decision cost function of HV can therefore be expressed as

(1) $$U^{HV}=\beta \cdot U_{te}^{HV}+(1-\beta )\cdot U_{ds}^{HV}+k_{Acc}\cdot U_{dc}^{HV}$$

where $U_{ds}^{HV},U_{te}^{HV}$, and $U_{dc}^{HV}$, respectively, represent the driving safety, travel efficiency, and driving comfort when HV adopts a specific strategy. β is an aggressiveness parameter that is used to represent specific driving styles. $k_{A}{}_{cc}$ is the driving comfort factor.

The cost of HV driving safety cost differs in the two situations of lane-keeping and lane-changing. When selecting a lane-keeping strategy, the safety cost is primarily related to the relative velocity and relative distance between the HV and the $PV_{\kappa }$ of the vehicle ahead in the current lane. The lateral safety cost between the HV and the target lane FV is primarily considered when the HV changes lanes [24]. The safety cost can be uniformly expressed as

(2) $$U_{ds}^{HV}=(1-|\gamma |)U_{ds-lk}^{HV}+|\gamma |U_{ds-lc}^{HV}$$

where $U_{lc}^{HV}$ and $U_{lk}^{HV}$ are the corresponding driving safety costs of HV lane changing and lane keeping, respectively. γ denotes the results of HV decision-making, i.e., γ∈{−1,0,1}:= {change to left lane, lane keeping, change to the right lane}.

$U_{lk}^{HV}$ is related to the relative distance and relative velocity of HV and $PV_{\kappa }$, which is expressed as

(3) $$U_{ds\text{-}lk}^{HV}=\left\{ \begin{array}{l} \psi_{v-lk}^{HV}\cdot \mathrm{sgn}[-\Delta V_{lk}^{PV_{\kappa }-HV}]\cdot {\left(\Delta V_{lk}^{PV_{\kappa }-HV}\right)}^{{}^{2}}\\ \;\;+\psi_{s-lk}^{HV}/[\Delta S_{lk}^{PV_{\kappa }-HV}+\varsigma ],if\exists PV_{\kappa }^{i}\\ \varpi_{lk},\;\;\;\;\;\;\;\;\;otherwise \end{array}\right.$$

where $PV_{\kappa }$ denotes the vehicle ahead in the current lane, and κ∈{1,2,3} indicates the lane ID of the HV. $\Delta V_{lk}^{PV_{\kappa }-HV}$ and $\Delta S_{lk}^{PV_{\kappa }-HV}$ are relative velocity and relative distance between HV and $PV_{\kappa }$. $\psi_{v-lk}^{HV}$ and $\psi_{s-lk}^{HV}$ are the respective weight coefficients for the velocity and distance terms. $\varpi_{lk}$ is a small number that represents the cost of selecting a lane-keeping strategy when the HV driving lane lacks $PV_{\kappa }$, and ς is a very small value to avoid a denominator of 0.

$U_{lc}^{HV}$ is related to the relative distance and relative velocity of HV and FV as follows:

(4) $$U_{\mathrm{ds}\text{-}\mathrm{lc}}^{HV}=\left\{ \begin{array}{l} \psi_{v-\mathrm{lc}}^{HV}\cdot \mathrm{sgn}[-\Delta V_{lc}^{HV-FV}]\cdot {\left(\Delta V_{lc}^{HV-FV_{}}\right)}^{{}^{2}}\\ \;\;+\psi_{s-\mathrm{lc}}^{HV}/[\Delta S_{lc}^{HV-FV_{}}+\varsigma ],\mathrm{if}\exists FV_{}\\ \varpi_{lc},\;\;\;\;\;\;\;\;otherwise \end{array}\right.$$

where FV represents the competing vehicle in the target lane, $\Delta V_{lc}^{HV-FV}$ and $\Delta S_{lc}^{HV-FV}$ are relative velocity and relative distance between HV and FV. $\psi_{v-\mathrm{lc}}^{HV}$ and $\psi_{s-\mathrm{lc}}^{HV}$ are the respective weight coefficients for the velocity and distance terms. $\varpi_{lc}$ is a small number that represents the cost of selecting a lane-changing strategy when the target lane lacks FV.

Driving comfort is mainly related to acceleration, which is defined by

(5) $$U_{dc}^{HV}=\psi_{acc}^{HV}{\left(\alpha_{x}^{HV}\right)}^{2}$$

where $\alpha_{x}^{HV}$ denotes the longitudinal acceleration of HV, and $\psi_{acc}^{HV}$ is the weight coefficient.

The travel efficiency of the HV is largely related to the longitudinal velocity. In this article, based on the research of P. Hang et al. [24] on the travel efficiency, the following further improvements are made. In this way, the HV has more initiative and flexibility when competing with other vehicles, and its travel velocity and space is significantly improved. $U_{\mathrm{te}}^{HV}$ can be given by

(6) $$U_{\mathrm{te}}^{HV}=\left\{ \begin{array}{l} {\left(\Delta V_{}^{v_{x}^{\max}-HV}\right)}^{2},\Delta s\geq d_{m}\\ {\left(\Delta V_{}^{PV_{\kappa }-HV}\right)}^{2},\Delta s<d_{m} \end{array}\right.$$

where $v_{x}^{\max}$ represents the maximum velocity allowed on the road, $d_{m}$ is the safe distance threshold at which the HV may accelerate without restriction, Δs is the relative distance between the host vehicle and the preceding vehicle. Under normal conditions, in response to the lane-changing behavior of HV, the competing vehicle will not accelerate to its maximum velocity to compete with the HV for the sake of driver safety. This paper therefore presumes that the maximum velocity that FV can achieve during the game is $0.8*v_{x}^{\max}$.

The cost function of FV has a similar structure to HV, which is not repeated here. The cost of driving safety is defined differently. Because FV does not take lane-changing behavior into account, its lateral driving safety mainly comes from the lane-changing behavior of HV. The lateral safety cost of FV is assumed to be equal to the lane-changing safety cost of HV, i.e., $U_{ds\text{-}lk}^{HV}=U_{ds\text{-}lat}^{FV}$.

### 3.3. Solution of the Game

Stackelberg game was raised by Heinrich Freiherr von Stackelberg in 1943 [25]. The total cost function of HV and FV are introduced into a Stackelberg game-based model, and the optimal strategy pairs are derived by solving the model. HV and FV play a 2-player Stackelberg game during the interaction. In contrast to the Nash game [26,27], the leaders HV and the follower FV are not independent and influence each other’s decisions. This is a typical two-layer optimization problem, which can be written as

(7) $$\left(\alpha_{x}^{HV*},\gamma^{*})=\underset{\alpha_{x}^{HV},\gamma }{argmin}(\underset{\alpha_{x}^{FV}\in \gamma^{2}(\alpha_{x}^{HV},\gamma )}{max}U^{HV}(\alpha_{x}^{HV},\gamma ,\alpha_{x}^{FV},\beta )\right)$$

(8) $$\gamma^{2}(\alpha_{x}^{HV},\gamma )=\{\sigma \in \gamma^{2}:U^{FV}(\alpha_{x}^{HV},\gamma ,\sigma ,\beta )\leq U^{FV}(\alpha_{x}^{HV},\gamma ,\alpha_{x}^{FV},\beta ),\forall \alpha_{x}^{FV}\in \gamma^{2}\}$$

(9) $$s.t.\gamma \in \{-1,0,1\},\gamma (\gamma +1)(\gamma -1)=0,\alpha_{x}^{HV}\in [\alpha_{x}^{\min},\alpha_{x}^{\max}],\alpha_{x}^{FV}\in [\alpha_{x}^{\min},\alpha_{x}^{\max}],$$

$$v_{x}^{HV}\in [v_{x}^{\min},v_{x}^{\max}],v_{x}^{FV}\in [v_{x}^{\min},v_{x}^{\max}]$$

where $U^{HV}$ and where $U^{FV}$ denote the total cost of HV and FV, and $\alpha_{x}^{HV*}$ is the optimal longitudinal acceleration of HV, $\alpha_{x}^{FV}$ is the longitudinal acceleration of FV, $\gamma^{*}$ is the optimal lane-changing command of HV, $\alpha_{x}^{\min}$ and $\alpha_{x}^{\max}$ are the minimum and maximum acceleration that a vehicle can reach, $v_{x}^{\min}$ and $v_{x}^{\max}$ are the minimum and maximum constraints of longitudinal velocity, $\gamma^{2}$ denotes the action candidates of FV, $\gamma^{2}(\alpha_{x}^{HV},\gamma )$ represents the optimal action candidates of FV given the actions of HV, β is the aggressiveness, $v_{x}^{HV}$ and $v_{x}^{FV}$ are the velocity of HV and FV.

For the bi-level optimization problem of the leader–follower game above, if a high-precision equilibrium solution is not required, the bi-level optimization problem can be solved by searching the discrete cost matrix extensively. The cost matrix is a finite composition, and it is easier to find an equilibrium. If the accuracy of the policy solution is very important, this bi-level optimization problem can also be solved by a bi-level genetic evolutionary algorithm (BLEAQ) [28]. It should be noted that, in order to satisfy the real-time calculation of decision-making, the optimal equilibrium of the game is calculated at every time step.

## 4. Controller Design Based on Driving Risk Field

### 4.1. Vehicle Kinematic Modeling

The motion planning of an AV includes longitudinal planning and lateral planning, and longitudinal planning refers to the velocity or acceleration planning of the AV in the longitudinal direction. In this paper, the lane-changing decision algorithm and the longitudinal planning of the vehicle are coupled into the decision-making module.

Lateral planning generally refers to path planning. To perform path-planning in real-time, an accurate model is required on the one hand, and the complexity of the model must be reduced on the other to reduce computational load and workload. To meet these two requirements, the following simplified vehicle kinematics model [29] is used in this paper.

(10) $$\chi =[v_{x}\;\varphi \;X\;Y{]}^{T}={\left[\alpha_{x}/v_{x},tan\beta /l_{r},\cos\theta /\cos\beta ,\sin\theta /\cos\beta \right]}^{T}\cdot v_{x}$$

(11) $$\beta =\arctan(l_{r}/(l_{r}+l_{f})\cdot tan\delta_{f})$$

(12) θ=φ+β

where the state vector $\chi ={\left[v_{x},\varphi ,X,Y\right]}^{T}$ and the control vector $u=\delta_{f}$. φ is the yaw angle. β and θ are the slip angle and heading angle, respectively. $\delta_{f}$ and (X,Y) are the steering angle of the front wheel and position coordinates. $\alpha_{x}$ and $v_{x}$ are the longitudinal acceleration (entered by the decision module) and velocity; $l_{r}$ and $l_{f}$ are the front and rear wheelbases, respectively.

To get the discretized system and for constraints of the control increment in the model predictive controller design hereinafter, Equation (10) should be transformed as follows:

(13) $$\xi (k+1)=\left[\begin{array}{l} v_{x}(k+1)\\ \varphi (k+1)\\ X(k+1)\\ Y(k+1)\\ \delta_{f}(k+1) \end{array}\right]=\left[\begin{array}{l} v_{x}(k)+\Delta t\cdot \alpha_{x}(k)\\ \varphi (k)+\Delta t\cdot v_{x}(k)tan\beta (k)/l_{r}\\ X(k)+\Delta t\cdot v_{x}(k)\cos\theta (k)/\cos\beta (k)\\ Y(k)+\Delta t\cdot v_{x}(k)\sin\theta (k)/\sin\beta (k)\\ \delta_{f}(k)+\Delta \delta_{f}(k+1) \end{array}\right]$$

where Δt is the discrete time step, and ξ(k+1) represents the state of the discrete system in the (k+1)th step. Δu=u(k)−u(k−1) and it represents $\Delta \delta_{f}$, while $\Delta \delta_{f}$ represents control inputs, which are the increments of vehicle front wheel steering angle in the kth step.

### 4.2. Driving Risk Field Modeling

To quickly quantify the driving risk level of vehicles in the road environment, based on the field theory, this paper establishes the driving risk field model of some necessary traffic entities, including obstacle vehicles, road boundaries, and lane lines. Among them, the paper considers the shape-size of the OVs and the different aggressiveness types. The risk level of these traffic entities is determined in detail by their own attributes and traffic regulations. Based on the vehicle kinematics model, MPC predicts the vehicle states and takes advantage of the time-efficient calculation and receding horizon optimization of the model. In this section, the risk level corresponding to the predicted position of HV is combined with the cost function of MPC to plan a collision-free lane-changing path for HV in real-time, and feedback on the states of the vehicle and the planned path parameters to the decision-making module.

#### 4.2.1. Risk Field of an Obstacle Vehicle

Considering a position (X, Y), the risk field model of surrounding obstacle vehicles can be given by [30]

(14) $$P_{ov}(X,Y)=A_{ov}\cdot \exp\left(-{\left(\frac{{\left(X\text{-}X_{ov}\right)}^{\varepsilon }}{{\left(k_{x}\cdot L_{ov}\right)}^{2}}+\frac{(X\text{-}X_{ov}{)}^{\varepsilon }}{{\left(k_{y}\cdot W_{ov}\right)}^{2}}\right)}^{\rho }\right)$$

where $A_{ov}$ is the maximum risk field value of OV, $\left(X_{ov},Y_{ov}\right)$ is the center of gravity coordinates of OV, ε are the coefficients to adjust the peak shape of the OV risk field, $L_{ov}$ and $W_{ov}$ are the length and width of OV, $k_{x}$ and $k_{y}$ are the adjustment coefficient of the OV’s shape size. ρ is the higher order coefficient Hereinafter, the strength of the risk field is represented by the character E.

**Figure 3:**
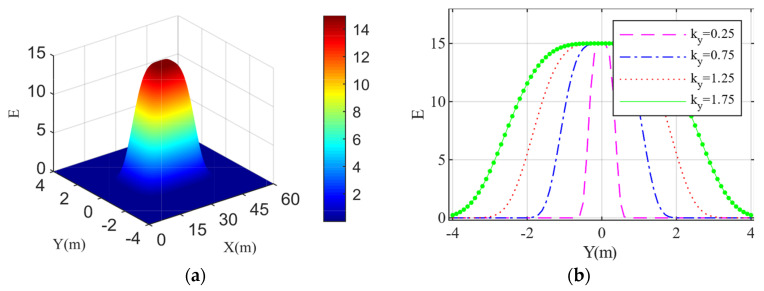
OV’s risk field: (**a**) 3D map of risk field; (**b**) Different coefficients of convergence of an OV’s lateral risk fields exerting on the HV.

The schematic diagram of the obstacle risk field is shown in Figure 3a. The smaller the relative distance between the HV and the OV, the greater the field strength of DRF at the HV’s position. Additionally, the risk field of the OV has a larger influence range in the longitudinal direction, and the risk field has a smaller influence range in the lateral direction, which is limited to the current driving lane. Under normal circumstances, an OV can occupy its own traffic lane, but cannot affect a vehicle in an adjacent lane, which has no relative displacement to it. According to Figure 3b, we set the optimal risk field convergence coefficient $k_{y}=0.75$ in the lateral direction of the OV.

**Figure 4:**
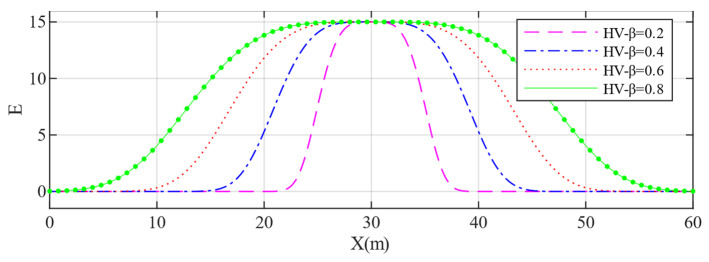
Risk field distribution for OV’s with different aggressiveness values along the X-axis.

Furthermore, different aggressiveness values have a greater impact on the risk field distribution of OVs, as shown in Figure 4. The figure shows that at the same velocity, the higher the aggressiveness value of the OV, the greater the influence range of the corresponding risk field in the driving direction reflected by different values of the $k_{x}$.

#### 4.2.2. Risk Field of an Obstacle Vehicle

The motion of the AVs should also be constrained by the road, including lane lines and road boundaries [31]. The risk field model of the road can be expressed as follows

(15) $$P_{Road}(X,Y)=\underset{P_{r1}}{\underset{︸}{A_{r1}\cdot \exp((-d_{r1}+d_{c}+W_{oc}/2)\cdot h_{1})}}+\underset{P_{r2}}{\underset{︸}{A_{r2}\cdot \exp(-d_{r2}^{2}/(\varepsilon \cdot \sigma_{line}^{2}))}}$$

where $P_{r1}$ and $P_{r2}$ are the field strength of the road boundary and lane lines at (X,Y) position, respectively. $d_{r1}$ and $d_{r2}$ are the minimum distance from the point (x,y) to the road boundary and lane lines. $d_{c}$ is the safety threshold. $W_{oc}$ denotes the OV’s width, $\sigma_{line}$, ε and $h_{1}$ are the risk field adjustment coefficients, respectively.

**Figure 5:**
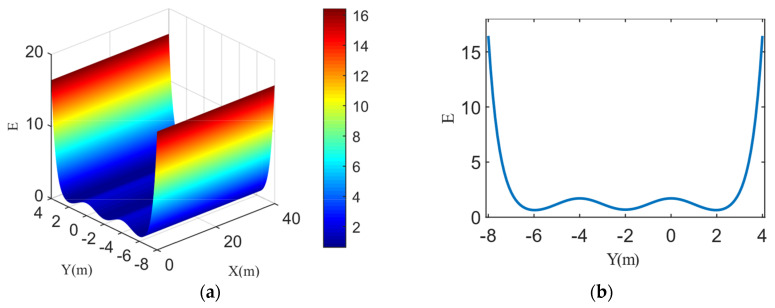
Road risk field: (**a**) 3D map of risk field; (**b**) A cross-sectional view along the Y-axis.

**Figure 6:**
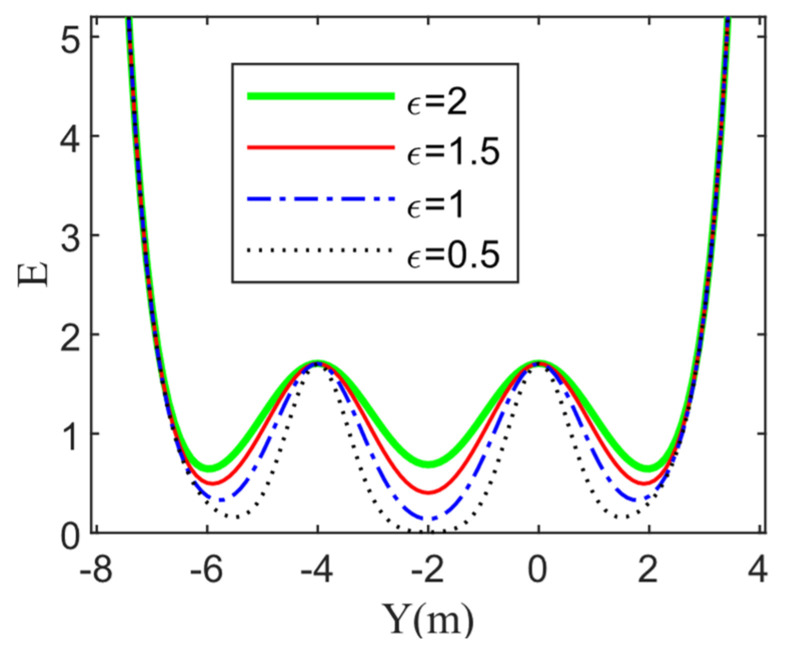
Different coefficients of convergence of the traffic line mark’s risk fields on the HV.

The three-lane road risk field is shown in Figure 5, where the left is a 3D map of the road risk field, and the right is the corresponding cross-sectional view along the Y-axis. The figure shows that the field strength near the road boundary is large, while the field strength in the free space of the road is very small. Moreover, the coefficient of convergence ε has a significant impact on the road risk field shown in Figure 6.

As discussed above, the DRF model needs to reflect various factors and build up a unified risk description of the surrounding environment of the host vehicle. The resulting DRF model is a combination of the obstacle vehicles and the road line marks, which are given as:

(16) $$P_{env}=\sum_{i=1}^{m}P_{ov(i)}+\sum_{j=1}^{n}P_{r1(j)}+\sum_{e=1}^{z}P_{r2(e)}$$

Where m is the number of obstacle vehicles, *n* and z are the number of road boundaries and lane lines, respectively, $P_{env}$ is the total risk value of a position.

**Figure 7:**
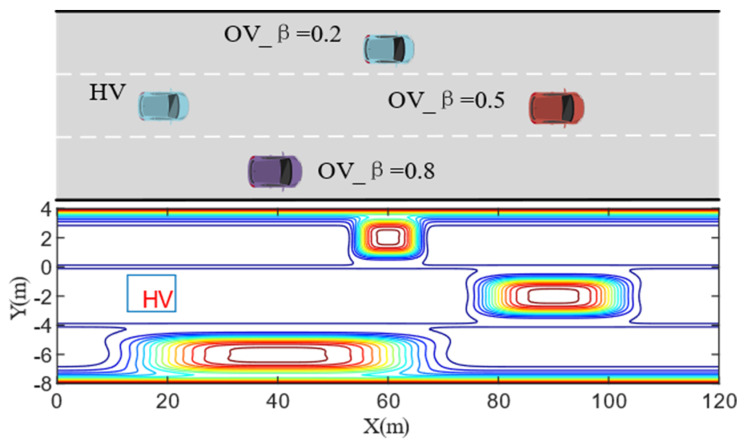
Contour distribution of HV’s driving risk field in the example scenario.

An example scenario with different kinds of aggressive OVs and HV, as well as the corresponding DRF contour maps is shown in Figure 7. The risk level of each location on the road can be visualized from the graph. Thus, the HV can efficiently calculate the safety level of each position on the road in complex traffic scenarios. On this basis, the HV can avoid high-risk areas and try to drive in low-risk level areas so as to make behavioral decision-making and path planning safely and efficiently improve travel efficiency. Notably, it also reflects the different levels of risk that different aggressiveness types vehicles can tolerate while driving.

### 4.3. DRF-Based MPC Controller

MPC is used in this paper to predict the motion state of HV for multiple steps in the future. The DRF strength of the predicted states of MPC is taken into account as a critical component of the MPC cost function to perform trajectory planning and motion control synchronously. The field strength of the HV’s kth prediction step is defined as follows:

(17) $$\Theta_{DRF}(k)=\Gamma [\chi (k),u(k)]=P_{env}(X(k),Y(k))$$

The MPC cost function should reflect the trajectory with not only the lowest driving risk but also the performance of adjusting the control increments for trajectory tracking and following the center-line of the target lane. Furthermore, to ensure the vehicle’s stability and comfort requirements, the front wheel steering angle should be constrained, so the control increments are also weighted as part of the cost function.

(18) $$\begin{array}{l} \min J(k)=\sum_{i=1}^{N_{p}}\left|\right|\Theta_{DRF}(i)|{|}_{Q_{1}}^{2}+\sum_{i=1}^{N_{p}}\left|\right|\Delta \varphi (i)|{|}_{Q_{2}}^{2}+\sum_{i=1}^{N_{p}}\left|\right|\Delta Y(i)|{|}_{Q_{3}}^{2}+\sum_{i=0}^{N_{c}-1}\left|\right|u(k+i|k)|{|}_{Q_{4}}^{2}\\ s.t.0\leq v\leq v_{\max},u_{\min}\leq u(k+i|k)\leq u_{\max},\Theta_{DRF}(k+i-1)=\Gamma [\chi (k+i-1|k),u(k+i-1|k)] \end{array}$$

where $\Theta_{DRF}(i)$ denotes the field strength in each step of the of the HV prediction horizon, the calculation method is defined by Equation (16); ΔY(i) and Δφ(i) are the lateral distance error and yaw angle error between the HV prediction position and the center-line of lane κ. Δu(k+i) represents the control increment which needs to be minimized. $Q_{1},Q_{2},Q_{3}$, and $Q_{4}$ are weighting factors of the optimization of the MPC controller, respectively. $N_{p}$ and $N_{c}$ represent the prediction horizon and the control horizon, respectively, $N_{p}\geq N_{c}$.

Equation (18) can be solved by a typical optimization algorithm [32], and the sequence of optimal control increments in the kth step can be obtained.

(19) $$\Delta u^{*}(k)=[\Delta u^{*}(k|k),\Delta u^{*}(k+1|k),\cdot \cdot \cdot ,\Delta u^{*}(k+N_{c}-1|k)]$$

Then, the control output at the time step k can be calculated.

(20) $$u(k|k)=u(k-1|k-1)+\Delta u^{*}(k|k)$$

Based on Equation (13), the state vector of HV can be updated, and at the next time step k+1, a new round of optimization is performed until the HV exits the game or successfully changes to the target lane.

## 5. Testing and Results Analysis

In this section, two typical highway lane-changing scenarios are designed to test the algorithm’s reliability and effectiveness. All algorithms and scenarios are implemented using MATLAB programs. Test scenario A is a two-lane highway scenario, which is designed to test the impact of the difference in the aggressiveness value of the two sides of the game on decision-making. Scenario B is an extension of the first scenario, and it attempts to test whether the same HV can make an appropriate lane-changing decision in a multi-vehicle game with various aggressive types of obstacle vehicles. This can be interpreted as reflecting the intelligence of the decision-making algorithm in this paper.

### 5.1. Parameters Setting

Table 2 shows the parameter settings of the decision model and the DRF model.

### 5.2. Test Scenario A

**Figure 8:**
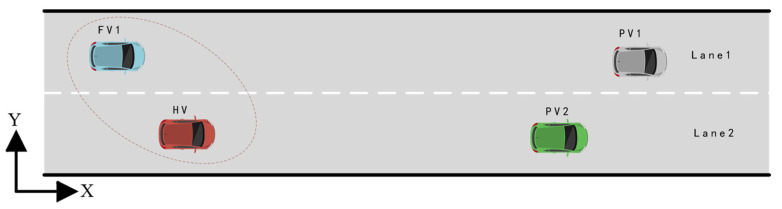
Testing Scenario A.

Scenario A examines a classic two-lane lane-changing scenario on expressways, as depicted in Figure 8. Because of the PV’s slow velocity, the HV must decide whether to change lanes or slow down to maintain a safe following distance from the PV2 in the current traffic environment. In order to better reveal the mechanism of aggressiveness types on game player decision-making, scenario A includes two cases. In case 1, FV1’s aggressiveness value is set to 0.6 (Moderate type) to analyze the effect of lane-changing decisions and path planning when HV with different aggressiveness levels play with FV1. In case 2, the aggressiveness value of FV1 is changed to 0.2 (conservative type), while the other settings keep unchanged. At the initial moment of the test, the position coordinates of HV, FV1, PV2, and PV1 are set as (8,−2), (6,2), (60,−2), and (100,2), respectively. The corresponding longitudinal velocities are 18 m/s, 10 m/s, 14 m/s, and 25 m/s. In order to simplify the test scenario, the velocity of PV2 and PV1 is assumed to be unchanged in both scenario A and scenario B.

**Figure 9:**
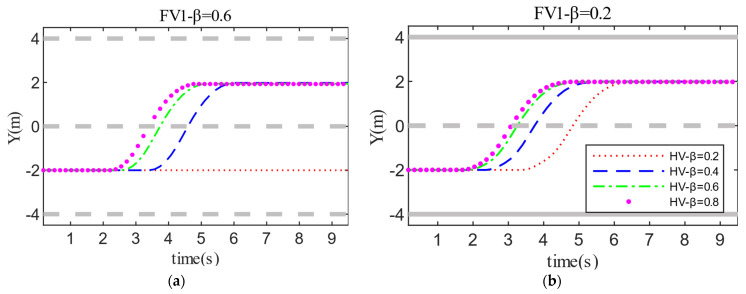
Test results for decision making and path planning in Scenario A: (**a**) FV1_β = 0.6; (**b**) FV1_β = 0.6.

**Figure 10:**
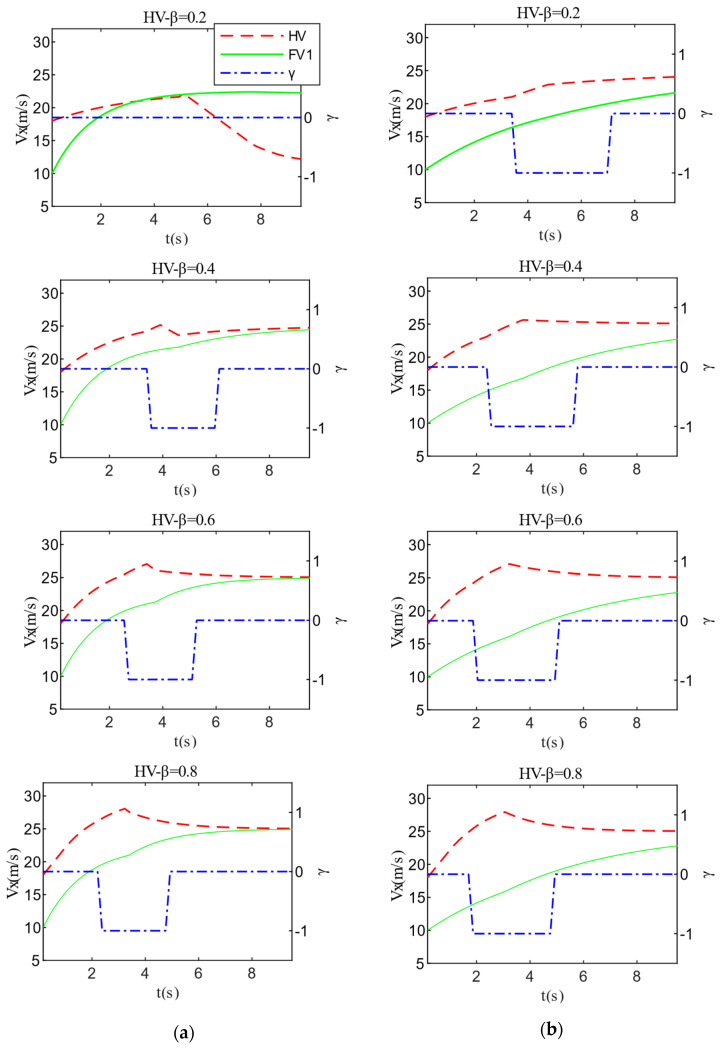
Velocity test results of Scenario A: (**a**) FV1_β = 0.6; (**b**) FV1_β = 0.2.

The detailed test results of the two cases in Scenario A are shown in Figure 9 and Figure 10. When the aggressiveness type of FV1 is fixed, changing the HV aggressiveness value has a greater impact on decision-making and path planning. For instance, when the HV’s aggressiveness is 0.2 and FV1’s aggressiveness is 0.6, the HV prioritizes driving safety when changing lanes and has lower velocity expectations. As a consequence, the HV was unable to change lanes successfully and finally chose the strategy of lane-keeping. Additionally, FV1 decelerates to keep a safe distance when approaching PV2. The increase in the aggressiveness value of the HV leads to a larger weight of the travel efficiency cost, so the evaluation of driving safety by the HV becomes bolder. The driving style of the HV also shifts from conservative to aggressive, which directly promotes the velocity and acceleration of the HV throughout the interaction. Another critical point is that the aggressiveness value of the HV is positively correlated with the time required to make a lane change decision. That is, the more aggressive the HV, the shorter the time it takes for the HV to make a lane-changing decision and complete the lane-changing process.

**Figure 11:**
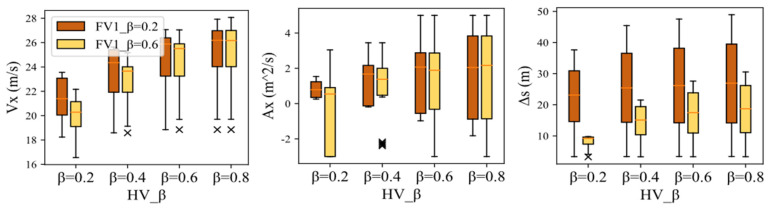
Box-plots for $V_{x}$, $A_{x}$, and ΔS in Scenario A.

Similarly, under the premise of the same HV aggressiveness, the different aggressiveness types of FV1 also have a certain degree of influence on HV decision-making. The reduction of the aggressiveness value causes FV1 to suppress the velocity expectation to ensure driving safety, which to some extent improves the dominant position of HV in the game process. Moreover, this also reflects that conservative FV1 hinders HV lane-changing behavior to a lesser degree than aggressive type. Therefore, the time required for the HV to make a lane-changing decision is shorter, and the acceleration and driving velocity are also improved. The test results in Figure 11 further validates the above analysis.

### 5.3. Test Scenario B

**Figure 12:**
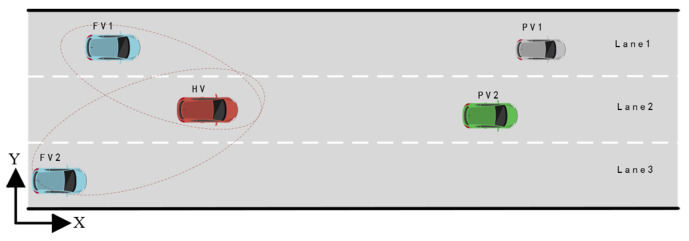
Testing Scenario B.

Scenario B is an upgrade of scenario A, as shown in Figure 12. Both HV and PV2 are driving in the middle lane, and PV2 is driving slowly. In this situation, the HV must choose whether to slow down or to change lanes. If the HV decides to change lanes, it must take into account the specific conditions of the competing vehicles in the target lane, such as aggressiveness, vehicle position, velocity and gap, etc. Then, the HV determines which target lane is less costly and risky to drive. In scenario B, the multi-vehicle game between HV and FVi (i = 1,2) needs to be considered. The positions of HV, FV1, FV2, PV2, and PV1 at the initial time are set to (8,−2), (6,2), (5,−6), (60,−2), and (95,2). The corresponding longitudinal velocities are 18 m/s, 10 m/s, 12 m/s, 14 m/s, and 26 m/s. Scenario B designs four cases to test whether HV can make rational and correct lane-changing decisions in the presence of diverse types of FVi (i = 1,2). In order to simplify scenario B and highlight the key points, this paper assumes that when HV decides to change lanes to the left, the game with FV2 ends immediately. Then, the velocity of FV2 remains unchanged while keeping the game with FV1 until the end of the lane change. Note that changing lanes to the right follows the same assumption.

**Figure 13:**
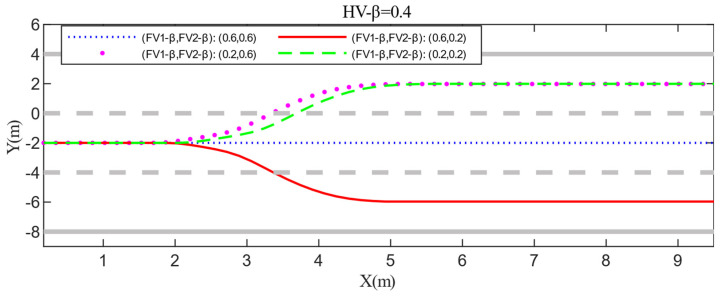
Test results for decision-making and path planning in Scenario B.

**Figure 14:**
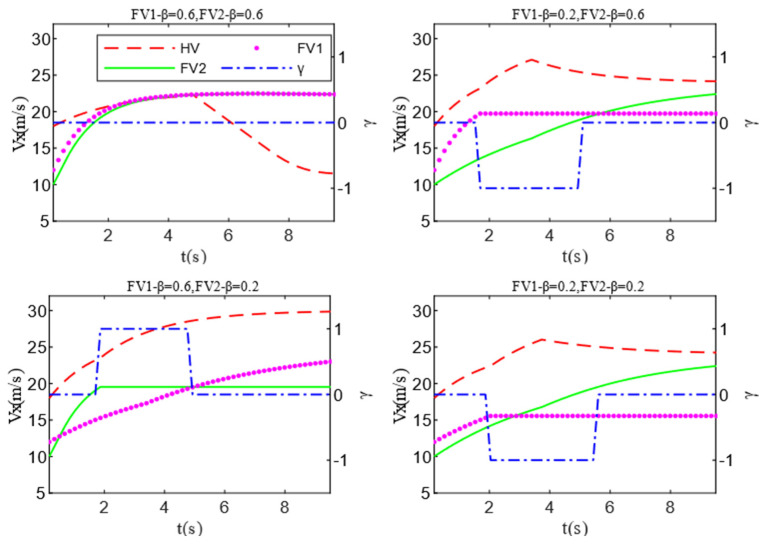
Velocity test results of Scenario B.

**Figure 15:**
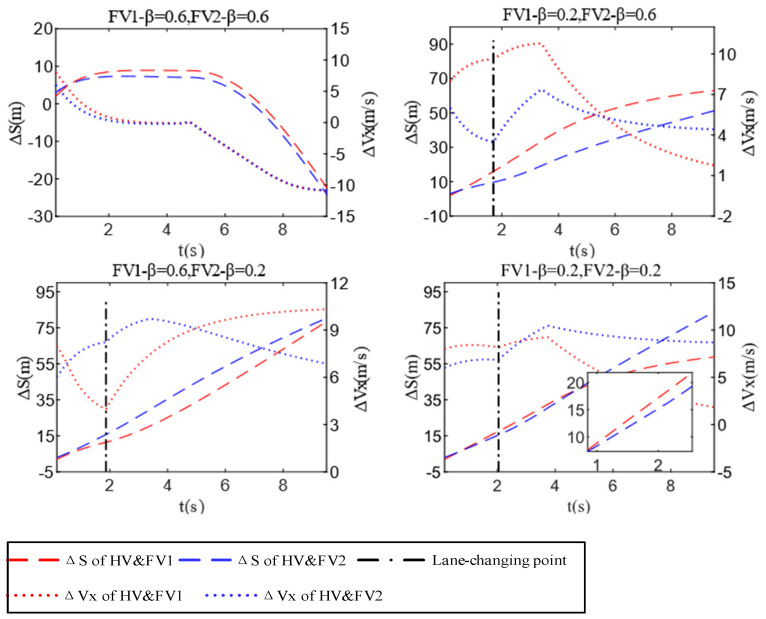
Test results of relative distance and relative velocity of scenario B.

The detailed test results of Scenario B are shown in Figure 13 and Figure 14. After summarizing, it can be discovered that because the positions and velocity of FV1 and FV2 are not significantly different, the difference in their aggressiveness types has a great impact on HV’s decision-making. For example, when the aggressiveness value of FV1 and FV2 are both 0.6, they will take a larger acceleration to prevent the HV from encroaching on their drivable space. In this situation, the HV has to stay in the lane to ensure driving safety and accelerate slowly to wait for the next opportunity to change lanes. When approaching PV2, slow down and follow it. If the aggressiveness types of FV1 and FV2 are not identical, the HV chooses the lane where the less aggressive obstacle vehicle is located when changing lanes. From the test results illustrated in Figure 15, it can be derived that the relative velocity and distance between HV and FVi (i = 1,2) increase as FV’s aggressiveness rise (HV minus FVi (i = 1,2)). This demonstrates that the advantages of velocity and drivable space markedly improved the HV’s initiative and safety when changing lanes while reducing the cost of making a decision. The last meaningful point is that if FVi (i = 1,2) have the same level of aggressiveness, then they hinder HV lane-changing behavior to the same degree. In this condition, the initial positions and velocities of FVi (i = 1,2) have obvious influence on the decision-making of HV. Case 4 of Scenario B proves this point.

## 6. Discussion

In this paper, the behavior decision algorithm is modeled using Stackelberg game theory, and then path planning and motion control are performed in real-time based on the decision result, with the vehicle states and path parameters fed back to the decision-making module. As a result, a closed-loop lane-changing decision-making framework that takes into account vehicle interaction and aggressiveness is designed. For the sake of improving the adaptability of the decision model for different driving groups, this paper incorporates the characteristics of different aggressive types into the design of the decision cost function and the DRF model. The effect of changes in aggressiveness value on decision-making and path planning is explored. Moreover, the DRF-based MPC controller developed in this paper can respond in real-time to decision-making commands and then plan a collision-free trajectory. The controller’s optimizer can be used to help determine the optimal path, which can then be followed by adjusting the control output simultaneously. Another advantage of this controller is that it can reduce computational load and improve real-time computing capability, which is critical for future AV applications.

Two typical highway scenarios are established in the simulation test to validate the algorithm in this paper. The results show that the closed-loop decision-making model can handle interactive behavior in complex traffic scenarios and make reliable lane-changing decisions intelligently. The algorithm has good application prospects.

Future work will further improve the decision-making algorithm based on game theory, primarily in terms of adaptability to more complex and heterogeneous traffic scenarios and dynamic recognition of vehicle aggressiveness types. In terms of algorithm validation, consider combining some experiments, such as driver-in-the-loop (MIL) and real-world vehicle verification, to more accurately evaluate and give feedback on the performance of the algorithm in this paper.

## Figures and Tables

**Table 1:** Game Formulation.

Decision Making		HV	
		Change Lanes $\left(\alpha_{lc\text{-}\min}^{HV},\alpha_{lc-\max}^{HV}\right)$	Stay $\left(\alpha_{lk-\min}^{HV},\alpha_{lk-\max}^{HV}\right)$
**FV**	$\left(\alpha_{\min}^{FV},\alpha_{\max}^{FV}\right)$	$\left(U_{lc}^{HV},U_{0}^{FV}\right)$	$\left(U_{lk}^{HV},U_{1}^{FV}\right)$

**Table 2:** Model parameters.

Stackelberg Game				DRF			
Parameter	Value	Parameter	Value	Parameter	Value	Parameter	Value
$\psi_{v-lk}^{HV}$	0.2	ς	1 × 10^−5^	ρ	2	$A_{r2}$	1.7
$\psi_{s-lk}^{HV}$	8 × 10^3^	$d_{m}$	20	$A_{obs}$	15	$L_{ov}$	4.4
$\psi_{v-lc}^{HV}$	0.2	$\alpha_{x}^{HV}$	[−3,5]	$A_{r1}$	1	$h_{1}$	2.2
$\psi_{s-lc}^{HV}$	8 × 10^3^	$v_{x}^{\max}$	30	$W_{ov}$	1.8	$d_{c}$	0.5
$\psi_{\alpha cc}^{HV}$	0.4	-	-	$\sigma_{line}$	10	ε	10

## Data Availability

Not applicable.
